# Evaluation of the Quality of Life of Patients with Myasthenia Gravis in Greece

**DOI:** 10.3390/jpm13071130

**Published:** 2023-07-12

**Authors:** Afrodite Aggelina, Eleftheria Karampli, Georgios Mavrovounis, Ioannis Boutsikos, Ioannis Pantazopoulos, Sotirios Kakavas, Elpida Pavi, Kostas Athanasakis

**Affiliations:** 1 Department of Public Health Policy, University of West Attica Athens, 115 21 Athens, Greece; afroaggel@gmail.com; 2 Laboratory for Health Technology Assessment, Department of Public Health Policy, School of Public Health, University of West Attica, 115 21 Athens, Greece; ekarabli@uniwa.gr (E.K.); epavi@uniwa.gr (E.P.); kathanasakis@uniwa.gr (K.A.); 3 Department of Emergency Medicine, Faculty of Medicine, University of Thessaly, 415 00 Larissa, Greece; boutsikosioannis@gmail.com (I.B.); pantazopoulosioannis@yahoo.com (I.P.); 4 ICU, Henry Dunant Hospital Center, 115 26 Athens, Greece; sotikaka@yahoo.com

**Keywords:** myasthenia gravis, quality of life, MG-QOL15r, patient-reported outcomes, neuromuscular disorders, MGQoL15, Greece

## Abstract

Myasthenia Gravis (MG) patients often report an affected quality of life (QoL). The aim of the current study was to evaluate the QoL of patients with MG in Greece using a specific tool. A cross-sectional online survey was performed. Adult patients were invited to participate. A questionnaire incorporating the MG-QOL15r scale was distributed, following its translation and cultural adaptation into Greek. Overall, 99 valid responses were submitted. The median age (interquartile range) of the participants was 48.50 (13.50) years and 76.80% were females. One third of the patients mentioned that they could not work/changed jobs after their diagnosis (28.30%) and that they face severe restriction of their everyday activities (26.30%). The mean MG-QOL15r score was 13.50 ± 7.70. Patients with important restriction of everyday activities (*p* < 0.01), patients with more pronounced need of emotional support (*p* < 0.01), patients with generalized MG (*p* < 0.01) and patients with myasthenic crises (*p* < 0.01) reported lower QoL. This study is the first to report on the affected QoL of the Greek population with MG using the MG-QoL15r scale. Further work should be done to incorporate the routine evaluation of QoL in the care of patients with MG.

## 1. Introduction

Myasthenia Gravis (MG) constitutes a chronic, autoimmune disorder with a step-wise progression of symptoms and involvement of multiple complex pathophysiologic pathways [1]. With an incidence of 0.3–2.8 per 100,000 people and a median prevalence of 10 per 100,000 people [2,3], it is the most common acquired disorder of the neuromuscular junction. MG is caused by the presence of autoantibodies against various components of the neuromuscular junction, usually against the acetylcholine receptor (AChR) [1,4]. The vast majority of patients (around 80%) have detectable antibodies against the AChR, while smaller percentages of patients have other antibodies, such as those against muscle-specific kinase or lipoprotein-receptor-related protein 4 [5]. The most commonly reported symptom in patients with MG is muscle weakness, which is especially pronounced at the end of the day. Importantly, extraocular muscles are affected causing diplopia and ptosis [6]. Apart from those symptoms, MG patients often exhibit a plethora of other symptoms and disorders, such as sleep disorders, cognitive, cardiac and autonomic dysfunction [1,2,3,4]. Treatment usually includes pyridostigmine and steroid-based immunosuppression; however, experts suggest individualization of the treatment plan for each patient [6].

Patients with MG are often unable to participate in their everyday activities in the same way that they did before their diagnosis, mainly due to the presence of significant muscle weakness [7]. In 2000, the Task Force of the Medical Scientific Advisory Board of the Myasthenia Gravis Foundation of America (MGFA), recognizing that patients with MG often report an affected QoL, advised in favor of the development of MG-specific tools for the evaluation of the QoL of patients with MG [8]. Indeed, in subsequent efforts to develop and validate such tools, patients with MG reported worse QoL when compared with non-diseased individuals [7,9]. Furthermore, various determinants of QoL have been identified, such as severity of symptoms and disease [10,11] and the presence of emotional disorders [12].

One of the most commonly implemented tools is the Myasthenia Gravis Quality of Life 15 (MG-QOL15) and its revised version (MG-QOL15r), developed by Neurologist Dr. Ted Burns and colleagues [9,13,14]. This tool has since been used and validated in various languages and has been established as one of the most important MG-specific tools for QoL assessment [15,16,17].

The aim of the current cross-sectional, questionnaire-based study was to investigate the effect of MG in the everyday lives of patients in Greece, using the MG-QOL15r tool for the first time, and to study the QoL of those patients.

## 2. Materials and Methods

The current study is a cross-sectional questionnaire-based study on the QoL of Greek patients with MG. The survey questionnaire included the MG-QOL15r scale (Greek version) and some additional demographic and disease-related questions. The collected data did not include any information that could be used to personally identify a participant.

There is no registry of MG patients in Greece; hence, the recruitment of participants was performed via the Hellenic Myasthenia Gravis Association (H-MGA). The study questionnaire was digitalized using the Google Forms platform and its distribution was performed via the H-MGA (18 October 2022) using their mailing list, numbering around 600 members (323 invitations). Members of the H-MGA received an email introducing the study and a link to the electronic questionnaire. The first page of the questionnaire provided information on the study objectives, the research team, data protection and mechanisms to ensure anonymity, the voluntary nature of participation and their right to withdraw at any time. Two follow-up emails were sent to increase the response rate (5 November 2022 and 25 November 2022).

This study was approved by the Research Ethics Committee of the University of West Attica (78607/7 September 2022) and the Board of Directors of the H-MGA (approved by H-MGA, 15 September 2022).

### 2.1. Inclusion Criteria

Patients were invited to voluntarily participate in this study if they fulfilled the following criteria: (1) members of the H-MGA with an active e-mail address, (2) ≥18 years old, (3) with a confirmed diagnosis of MG.

### 2.2. Questionnaire

The questionnaire used in the current study was developed based on the available literature on the topic and included the following sections: demographics, clinical characteristics, general questions on patients’ everyday life and QoL, and MG-QOL15r (Greek version). The questions are presented in Supplementary Table S1.

### 2.3. Translation and Cultural Adaptation of the MG-QOL15r Scale

The MG-QOL15r scale is a standardized tool, translated in 11 languages, that facilitates the study of the QoL in patients with MG. Permission to use the questionnaire was obtained from the author via email. In general, greater scores indicate a greater disease impact on everyday life. The translation and cultural adaptation of the questionnaire for use in the Greek population followed the principles suggested by the Wild et al. study in 2005 [18]. The process followed for translation and cross-cultural adaptation is briefly described below:

Two independent researchers (native Greek speakers) familiar with medical terminology and with MG translated the questionnaire from English to Greek (forward translation). The two translations were then compared and merged into one (reconciliation), making sure that content validity was maintained in the process and that appropriate adjustments were made to culturally adapt the questions. In turn, the translated questionnaire was backward translated into English. All translations were then studied by the researchers, and a pre-final version of the MG-QOL15r questionnaire in the Greek language was created. The pre-final version was used in interviews with a sample of five participants chosen to be representative of the target population (purposive sampling). When asked, 100% of the participants rated the questions as easily comprehensible, relevant to the disease, and would not change anything in the wording of the questions, establishing face validity (cognitive debriefing). Finally, the final Greek version of the MG-QOL15r was created.

### 2.4. Data Curation and Statistical Analysis

During questionnaire cleaning, we excluded all questionnaires from participants who did not answer the MG-QOL15r subsection in its entirety. The data were then coded and imported into the statistical analysis software. Statistical analysis was performed using the IBM Corp. Released 2017. IBM SPSS Statistics for Macintosh, Version 25.0. Armonk, NY, USA: IBM Corp. Continuous data were checked for normality using the Shapiro–Wilk test. Qualitative variables are presented as percentages. Continuous data with normal distributions are presented as means ± standard deviation (SD) and comparisons between groups were performed using unpaired two-tailed *t*-tests and analysis of variance (ANOVA), as appropriate. Post hoc analysis was performed using the Tukey test. Data with non-normal distributions are presented as medians (interquartile range—IQR), and comparisons between groups were performed using the Kruskal–Wallis H test with post hoc analysis. Cronbach’s alpha was calculated to investigate the internal consistency of the translated questionnaire. Finally, the Spearman’s Rank Correlation Coefficient was used to elucidate the correlation between variables.

## 3. Results

Overall, we collected 123 responses generating a response rate of 38.10% (323 invitations). After questionnaire cleaning, 99 questionnaires were included for analysis.

### 3.1. Demographic Data and Everyday Life

The median age (IQR) of the participants was 48.50 (13.50) years. Regarding the gender, 22 (22.20%) patients were males and 76 (76.80%) were females, while 1 patient elected not to provide any gender information. More than half of the participants (*n* = 57, 57.60%) reported that they are married, and 62 (62.60%) reported that they have children. On their living situation, most of the participants 73 (73.70%) reported that they live with at least another person in the same house.

The vast majority of patients (*n* = 78, 78.80%) reported that they are insured with the National Social Security Fund. Around one-third of the participants (*n* = 34, 34.30%) reported that they are retired. Fifty-three (53.50%) patients resided in the Attica region. Regarding the impact of MG on their employment status, 28 participants (28.30%) reported that they either cannot work or were forced to change jobs as a consequence of their disease. In addition, 22 (22.20%) participants retired due to disability. With regard to the household economic status, 35 (35.40%) participants replied that they face “moderate” financial challenges, whereas 33 (33.30%) participants mentioned that they face “severe” or “very severe” financial challenges in their household. The majority (39.40%) of participants replied that the healthcare costs associated with MG had a “minor” effect on their household economic status. However, 18 (18.20%) participants mentioned an “Important” and “Very important” effect of MG-related healthcare costs on their household economic status.

When asked about their everyday life, 53 (53.50%) and 26 (26.30%) patients reported mild and severe restriction of their everyday routine, respectively. Only one patient reported an inability to perform any activity without help. More than half of the participants (*n* = 14/27, 51.90%) that reported “severe restriction of their everyday routine” or “complete inability to perform activities without help” mentioned that they receive help from someone who lives in the same household. Importantly, when asked about getting help with their day-to-day activities from a neighbor, the vast majority of the participants replied that it is difficult (*n* = 29, 29.30%) or very difficult (*n* = 33, 33.30%). Finally, 67 participants (67.70%) reported that during the last year they felt a greater need for emotional support when compared to the past. Table 1 presents the main demographic data.

### 3.2. Health-Related and Disease-Specific Data

On their general health status, most participants reported that they would describe their health as “moderate” (*n* = 40, 40.40%) or “good” (*n* = 32, 32.30%). The median (IQR) reported score of their health on a scale from 0 to 100 was 67 (30); 54 (54.50%) reported that they have other chronic health conditions (duration >6 months) in addition to MG. Health-related data are presented in Table 2.

Most participants (*n* = 86, 86.90%) reported that they have a diagnosis of generalized MG, and the median (IQR) time from diagnosis was 13 (13.50) years. Interestingly, only around one third (*n* = 34, 34.30%) of the participants reported myasthenic crises as part of their disease. However, 58 (58.60%) of the participants reported that they have a greater than 50% disability as a result of having MG. The breakdown of the most commonly reported symptoms is presented in Table 3.

### 3.3. MG-QOL15r

Cronbach’s alpha for internal consistency was found to be 0.94, indicating a high level of internal consistency. The mean reported MG-QOL15r score was 13.50 ± 7.70. Patients who reported no or minimal change in their everyday routine reported a statistically significant (*p* < 0.01) lower mean MG-QOL15r score (mean ± SD: 10.80 ± 6.60) when compared to those that reported severe change or inability to perform their everyday routine (mean ± SD: 20.70 ± 5.50). Furthermore, participants who reported an increased need for emotional support during the last year had a statistically significant (*p* < 0.01) higher MG-QOL15r score (mean ± SD: 15.10 ± 7.60) when compared to those that did not (mean ± SD: 10.10 ± 6.90). Patients with ocular MG (mean ± SD: 5.80 ± 5.40) reported significantly lower (*p* < 0.01) MG-QOL15r scores when compared to those with generalized MG (mean ± SD: 14.60 ± 7.30). Patients without episodes of myasthenic crises (mean ± SD: 11.20 ± 7.30) reported significantly lower (*p* < 0.01) MG-QOL15r scores when compared to those with myasthenic crises (mean ± SD: 17.10 ± 6.80).

A statistically significant difference (*p* < 0.01, H: 22.50) was also identified between patients that reported different general health statuses. More specifically, we identified a mean rank MG-QOL15r score of 33 for the “very good” and “good” health group, 58.50 for the “medium” health group, and 64.50 for the “poor” and “very poor” health group. A post hoc analysis revealed statistically significant differences (*p* < 0.01) in the comparisons between the “very good” and “good” health group vs. the “medium” health group, and the “very good” and “good” health group vs. the “poor” and “very poor” health group. This was also evident in the negative correlation between the numerical value that participants assigned to their health status (0–100) and the MG-QOL15r score (Rs: −0.60, *p* < 0.01). As expected, those with greater than 50% disability (mean ± SD: 15.70 ± 6.50) reported significantly (*p* < 0.01) higher MG-QOL15r scores compared to those without (mean ± SD: 10.10 ± 8.60). Finally, the presence of financial challenges was also associated with a statistically significant difference (*p* < 0.01, H: 17.70) in the reported MG-QOL15r score; similarly, participants that reported a greater effect of MG-related expenses on their financial status also reported statistically (*p* < 0.01, F: 6.40) higher MG-QOL15r scores.

## 4. Discussion

In this questionnaire-based study, we identified a significant effect of MG in the everyday QoL of patients. Overall, the vast majority of patients reported mild or severe restriction of their everyday routine, indicating that they need to receive help from someone else to perform their day-to-day activities. Most patients did not report active myasthenic crises as part of their disease, although they reported greater than 50% disability as a result of MG. Using the MG-QOL15r scale, we identified a lower reported QoL in patients that reported important restriction of their everyday activities, in patients with a greater need of emotional support and in those with generalized MG in contrast to ocular MG. As expected, patients who experience myasthenic crises as part of their disease reported worse QoL than those who do not. Finally, it is notable that patients who reported a greater effect of MG-related costs in their financial status also reported a worse QoL.

In line with our results, previous large studies from around the globe have demonstrated that MG patients report a low quality of life with an increased need for emotional, financial and pharmacological support [5,6,7]. The MG-QOL15 and MG-QOL15r instruments have been widely used in the MG-related literature in both everyday clinical practice and research-related efforts [8,9,10,11]. More specifically, they have been used for obtaining information regarding patients’ perspective on their disease [8], for following up patients [12,13], for studying the QoL of patient cohorts [14] and for comparing cohorts of patients with MG in clinical trials [15]. The mean MG-QOL15r score ± SD (13.50 ± 7.70) reported in our study is similar to that of previous studies using the same questionnaire in other populations with different geographical and cultural backgrounds [9,16,17]. The association between MG and the mood of patients is widely reported in the literature, as several studies have reported worse QoL in patients with self-reported depression symptoms [18,19]. This was also the case in our study as patients with an increased need for emotional support reported worse QoL (mean MG-QOL15r score ± SD: 15.10 ± 7.60) in comparison to those without an increased need for emotional support (mean MG-QOL15r score ± SD: 10.10 ± 6.90). Healthcare providers should keep this in mind and are advised to screen patients with MG for mood disorders. Cultural differences in seeking help and expressing weakness should be kept in mind and are discussed below. Furthermore, as expected, the worse QoL reported by patients with generalized MG in comparison with patients with ocular MG in our study has also been previously reported [14]. Interestingly, a previous study from China identified an association between female gender and worse self-reported QoL. The authors suggested several hormone-related and pharmacokinetics-related factors as potential explanations [20]. This association was not replicated in our study.

In our study, as in previous large studies [21,22], substantial percentages of patients reported significant effects of MG on their employment and income status, leading to worse QoL. Moreover, our findings seem to agree with other studies on QoL using different questionnaires. More specifically, it has been previously reported that the severity of symptoms and the frequency of myasthenic crises are associated with worse QoL, as identified by the Short Form-36 (SF-36) questionnaire [23]. Also, our results are in accordance with previous studies concerning the medical expenses of MG patients. Those with higher MG-related expenses reported worse scores in SF-36 questionnaire-based studies [22].

It is important for healthcare professionals who are involved in the care of patients with MG to inform the patients of the various modifiable factors that could improve their disease and everyday QoL. Patients should be advised to follow a proper handwashing routine, to stop smoking and to follow the age-appropriate vaccination recommendations [24]. These interventions could reduce the frequency of infections which in turn can precipitate myasthenic crises [24,25]. Furthermore, the patients should be educated on drugs that could worsen MG symptoms, for example, several antibiotic categories such as macrolides and aminoglycosides [26].

As previously mentioned, another important concept that should be kept in mind by healthcare professionals treating patients with MG and researchers evaluating the QoL of those patients is the cultural differences between different populations. As indicated by Jeong et al. in a study on the Korean population, in regions where “face saving” culture is prominent and seeking help might be perceived as a weakness [27], patients might avoid discussing their physical or mental symptoms [28]. A similar consideration was highlighted in a systematic review of research on quality of life in the health sciences and medicine. In that study, the authors underline that different people from different cultures might understand the concept of QoL differently [29]. Another important consideration to be mentioned is that in Greece, where strong family bonds are maintained throughout life [30], emotional support, help with everyday tasks and even economic support might be easier to obtain.

There are some limitations to our study that should be noted. Our sample size was small when compared to other studies from countries with larger populations; also, due to the lack of a national registry of MG patients, ours is a convenience sample of MG patients in Greece. Nevertheless, our study is the first to provide data on the impact of MG on the QoL of patients in Greece using a validated instrument. Furthermore, the data were self-reported by participants, not in an inpatient or outpatient hospital setting. Consequently, it was not possible to collect more specific clinical information regarding the participants. Therefore, it was not possible to confirm details of clinical diagnoses and medical history and correlate patient reported data to clinically assessed measures such as the MGFA classes of disease, which possibly introduces some bias (e.g., reporting bias) to our analyses. This, in turn, highlights an important consideration inherent in most patient-reported outcome measures, such as the MG-QoL15r questionnaire, which is the fact that patient-reported symptoms and disease manifestations should always be accompanied by clinical evaluation, especially in everyday clinical work. Another consideration associated with the questionnaire used is that QoL can be affected by factors not directly related to MG. These factors could have been missed by the implemented scale. The web-based nature of the study might have caused some selection bias in favor of younger participants and participants with access to a computer and an Internet connection. Finally, it should be mentioned that the addition of further patient-reported outcome measures and other scales in the present work would have enabled us to extract more robust results; however, we elected to only include one scale in our project to enhance the potential of a higher response rate [31].

## 5. Conclusions

In conclusion, our findings emphasize that Greek MG patients in the present study struggle in their everyday life due to either the disease severity or their perspective concerning their condition. The MG-QOL15r questionnaire is a helpful clinical and research tool for the evaluation and follow-up of patients with MG. Continuous effort towards the improvement of medical, pharmacological and social management, via specific care pathways, will contribute to the actual improvement of patients’ QoL.

## Figures and Tables

**Table 1:** Table presenting the main demographic information of participants.

Question (Q)	Answer Choices	Responses—N (%)	MG-QOL15rscore [Mean ± SD; Median(IQR)] or Correlation between MG-QOL15r Score and Variable (r_s_)	*p* Value
Q20. Biological sex	a. Male b. Female c. I do not wish to answer	22 (22.20) 76 (76.80) 1 (1)	a: 12.10 ± 7.50 b: 14.10 ± 7.60	0.29
Q21. Year of birth	Date	Median age (IQR): 48.50 ± 16	0.005	1
Q22. Marital status	a. Never married b. Married c. Divorced/separated d. Widowed e. I do not wish to answer	30 (30.30) 57 (57.60) 7 (7.10) 5 (5.10) -	a + c + d: 13.40 ± 8.20 b: 13.50 ± 7.40	0.92
Q23. How many children do you have?	Open response	Median number (IQR): 1 (2)	Have children: 14.10 ± 7.50 No children: 12.50 ± 8.10	0.31
Q24. How many people do you currently live with?	Open response	Median number (IQR): 2 (1)	Living with others: 14.10 ± 7.50 Living alone: 11.90 ± 8.10	0.21
Q25. Select your educational level	a. I have not finished primary school b. Primary school c. Secondary school d. High school e. College/Vocational training f. University g. Postgraduate studies h. I do not know/I do not wish to answer	- 2 (2) 2 (2) 29 (29.30) 17 (17.20) 34 (34.30) 15 (15.20) -	b + c + d: 13.90 ± 8.30 e + f + g: 13.20 ± 7.40	0.70
Q26. In which prefecture are you currently living in?	a. Open response b. I do not wish to answer	Attica: 53 (53.50) Rest of Greece: 38 (38.40) I do not wish to answer: 8 (8.10)	Attica: 13.20 ± 7.60 Rest of Greece: 14.20 ± 8.30	0.50
Q27. What is your employment status?	a. Self-employed b. Employee c. Working without compensation in the family business d. Retired e. Unemployed f. Student/Housework g. Other h. I do not wish to answer	16 (16.20) 33 (33.30) 2 (2) 34 (34.30) 10 (10.10) 2 (2) 1 (1) 1 (1)	-	-
Q28. Which of the following better described your insurance coverage?	a. Public b. Private c. Private and public d. No insurance e. Coverage for indigent people f. I do not wish to answer	78 (78.80) 4 (4) 13 (13.10) 3 (3) - 1 (1)	Mean rank a: 51.20 b: 91.50 c: 29.70 d: 34.70	<0.01
Q30. Which of the following better describes the economic status of your household?	a. Very severe financial challenges b. Severe financial challenges c. Some financial challenges d. Good financial status e. Very good financial status f. Excellent financial status g. I do not know/I do not wish to answer	9 (9.10) 24 (24.20) 35 (35.40) 18 (18.20) 10 (10.10) 2 (2) 1 (1)	Mean rank a + b: 65.10 c + d: 44.40 e + f: 29.40	<0.01

Abbreviations: MG-QOL15r—revised MG-QOL15; SD—standard deviation; IQR—interquartile range.

**Table 2:** Table presenting the main health-related information of participants.

Question (Q)	Answer Choices	Responses—N (%)	MG-QOL15r Score [Mean ± SD; Median(IQR)]	*p* Value
Q1. How would you describe your everyday life with MG?	a. No change in everyday routine b. Mild restriction in everyday routine c. Severe restriction of everyday routine d. Inability to perform everyday routine without help	19 (19.20) 53 (53.50) 26 (26.30) 1 (1)	a + b: 10.80 ± 6.60 c + d: 20.70 ± 5.50	<0.01
Q2. If you answered “c” or “d” in Q1, who provides help with your everyday routine?	a. Someone who lives with me—household member b. Partner or relative who does not live with me c. Someone who does not live with me and is not compensated for help (e.g., neighbor, friend, colleague) d. Someone who is paid for help by me e. Someone in the context of an organized help program f. Other g. I do not wish to answer a + b a + c	14/27 3/27 3/27 3/27 1/27 - 1/27 1/27 1/27	-	-
Q3. How would you rate the level of interest of others in your everyday life?	a. None b. Minimal c. Moderate d. High e. I do not know/I am not sure f. I do not wish to answer	12 (12.10) 23 (23.20) 33 (33.30) 23 (23.20) 5 (5.10) 3 (3)	a + b: 12.50 ± 7.00 c + d: 14.10 ± 8.30	0.35
Q4. How easy is it to obtain help from your neighbor/s if needed?	a. Very difficult b. Difficult c. Possible d. Easy e. Very easy f. I do not wish to answer	33 (33.30) 29 (29.30) 26 (26.30) 5 (5.10) 4 (4) 2 (2)	a + b: 14.10 ± 7.30 c: 13.10 ± 8.40 d + e: 10.20 ± 9.00	0.37
Q5. How many people can you count on when facing important personal issues?	a. 0 b. 1–2 c. 3–5 d. 6+ e. I do not know/I do not wish to answer	2 (2) 45 (45.50) 39 (39.40) 13 (13.10) -	a: 18.00 ± 4.20 b: 14.80 ± 7.90 c: 11.50 ± 6.80 d: 14.00 ± 8.90	0.20
Q6. In the last year, did you need more emotional support when compared to the past?	a. Yes b. No c. I do not know/I do not wish to answer	67 (67.70) 31 (31.30) 1 (1)	a: 15.10 ± 7.60 b: 10.10 ± 6.90	<0.01
Q29. Has your MG diagnosis affected your employment status?	a. No b. Decreased hours c. Changed job d. I am not able to work due to MG e. Retired due to disability	27 (27.30) 14 (14.10) 12 (12.10) 16 (16.20) 22 (22.20)	Mean rank a: 28.50 b: 49.40 c: 41.30 d: 67.40 e: 52.30	*p* < 0.01
Q31. During the last 12 months, how greatly do you believe that the healthcare costs associated with MG affected your household economic status?	a. No effect b. Minor effect c. Some effect d. Important effect e. Very important effect f. I do not know g. I do not wish to answer	14 (14.10) 39 (39.40) 24 (24.20) 11 (11.10) 7 (7.10) 1 (1) 3 (3)	a + b: 11.60 ± 7.30 c: 15.20 ± 7.30 d + e: 18.30 ± 7.00	<0.01

Abbreviations: MG—Myasthenia Gravis; MG-QOL15r—revised MG-QOL15; SD—standard deviation; IQR—interquartile range.

**Table 3:** Table presenting the main disease-specific information of participants.

Question (Q)	Answer Choices	Responses—N (%)	MG-QOL15r Score [Mean ± SD; Median (IQR)]	*p* Value
Q7. How would you rate your general health status at the moment?	a. Very bad b. Bad c. Moderate d. Good e. Very good f. I do not know/I do not wish to answer	5 (5.10) 14 (14.10) 40 (40.40) 32 (32.30) 7 (7.10) 1 (1)	Mean rank a + b: 64.50 c: 58.50 d + e: 33.00	<0.01
Q8. Rate your health on a scale of 0–100 (0: near death, 100: excellent)	0–100	Median (IQR): 67 (30)	−0.60	<0.01
Q10. When did you receive your MG diagnosis? (year)	Date	Median time from diagnosis in years (IQR): 13 (13.50)	−0.15	0.16
Q11. I suffer from	a. Ocular myasthenia b. Generalized myasthenia	13 (13.10) 86 (86.90)	a: 5.90 ± 5.40 b: 14.60 ± 7.30	<0.01
Q12. If you answered “b” in the previous question, choose the stage of the disease that you are currently at	a. Initial b. Second c. Third d. Terminal e. I do not know f. I do not wish to answer	7 (7.10) 8 (8.10) 17 (17.20) 5 (5.10) 46 (46.50) 3 (3)	a: 11.70 ± 9.50 b: 11.50 ± 6.60 c: 18.80 ± 5.40 d: 22.80 ± 5.40	<0.01
Q13. Which of the following is/are the most common symptom/s that you experience?	a. Muscle weakness b. Blepharoptosis c. Easy fatigue d. Respiratory distress e. Diplopia f. Other	75 (75.80) 44 (44.40) 79 (79.80) 34 (34.30) 44 (44.40) 8 (8.10)	-	-
Q14. Do you have myasthenic crises?	a. Yes b. No c. I do not know/I do not wish to answer	34 (34.30) 60 (60.60) 5 (5.10)	a: 17.10 ± 6.80 b: 11.20 ± 7.30	<0.01
Q15. If you answered “a” in the previous question, how many episodes did you have in the last 3 months?	a. Number b. I do not know/I do not wish to answer	0 episodes: 13/34 1 episode: 10/34 2 episodes: 3/34 4 episodes: 2/34 9 episodes: 1/34 15 episodes: 1/34 20 episodes: 1/34 25 episodes: 2/34 I do not know/I do not wish to answer: 1/34	-	-
Q16. Do you take any medications for MG?	a. Yes b. No c. I do not wish to answer	94 (94.90) 5 (5.10) -	-	-
Q17. If you answered “a” in the previous question, choose the appropriate answer	a. Only pyridostigmine b. Pyridostigmine and cortisol c. Pyridostigmine, cortisol and an immunosuppresant d. Other e. I do not know/I do not wish to answer	24/94 22/94 36/94 12/94 -	a: 12.00 ± 7.60 b: 11.70 ± 8.00 c: 15.40 ± 6.90 d: 14.70 ± 7.40 No drug: 12.00 ± 12.10	0.30 (also no statistically significant difference between those that were receiving and not receiving cortisol)
Q18. Do you have any other chronic health conditions? (>6 months)	a. Yes b. No c. I do not know/I do not wish to answer	54 (54.50) 38 (38.40) 7 (7.10)	a: 14.60 ± 7.20 b: 11.50 ± 8.20	0.05
Q19. Do you have a disability percentage greater than 50% as a result of MG?	a. Yes b. No c. I do not wish to answer	58 (58.60) 34 (34.30) 7 (7.10)	a. 15.70 ± 6.50 b. 10.10 ± 8.70	<0.01

Abbreviations: MG—Myasthenia Gravis; MG-QOL15r—revised MG-QOL15; SD—standard deviation; IQR—interquartile range.

## Data Availability

The participants of this study did not give written consent for their data to be shared publicly, so due to the sensitive nature of the research, supporting data are not available.

## Supplementary Materials

The following supporting information can be downloaded at: https://www.mdpi.com/article/10.3390/jpm13071130/s1, Table S1: Table presenting the breakdown of the questionnaire used for the study.
