# Supplementary material for: Evaluation of the Quality of Life of Patients with Myasthenia Gravis in Greece
Source: J Pers Med. 2023 Jul 12;13(7):1130. doi: 10.3390/jpm13071130 (PMC10381620; doi:10.3390/jpm13071130)
Supplement: Supplementary file 1 [file jpm-13-01130-s001.zip › jpm-2493744-supplementary.pdf]

Table S1. Table presenting the breakdown of the questionnaire used for the study.

Question (Q)	Answer choices
1. How would you describe your everyday life with MG?	a. No change in everyday routine<br>b. Mild restriction in everyday routine<br>c. Severe restriction of everyday routine<br>d. Inability to perform everyday routine without help
2. If you answered "c" or "d" in Q1, who provides help with your everyday routine?	a. Someone who lives with me - houshold member<br>b. Partner or relative who does not live with me<br>c. Someone who does not live with me and is not compensated for help (e.g. neighbor, friend, colleague)<br>d. Someone who is paid for help by me<br>e. Someone in the context of an organized help program<br>f. Other<br>g. I do not wish to answer
3. How would you rate the level of interest of others in your everyday life?	a. None<br>b. Minimal<br>c. Moderate<br>d. High<br>e. I do not know/ I am not sure<br>f. I do not wish to answer
4. How easy is it to obtain help from your neighbor/s if needed?	a. Very difficult<br>b. Difficult<br>c. Possible<br>d. Easy<br>e. Very easy<br>f. I do not wish to answer
5. How many people can you count on when facing important personal issues?	a. 0<br>b. 1-2<br>c. 3-5<br>d. 6+<br>e. I do not know/ I do not wish to answer
6. In the last year, did you need more emotional support when compared to the past?	a. Yes<br>b. No<br>c. I do not know/ I do not wish to answer
7. How would you rate your general health status at the moment?	a. Very bad<br>b. Bad<br>c. Moderate<br>d. Good<br>e. Very good<br>f. I do not know/ I do not wish to answer
8. Rate your health on a scale of 0-100 (0: near death, 100: excellent)	0-100
9. 15 questions of the MGQoL15-R questionnaire	
10. When did you receive your MG diagnosis? (year)	Date
11. I suffer from	a. Ocular myasthenia<br>b. Generalized myasthenia
12. If you answered "b" in the previous question, choose the stage of the disease that you are currently at	a. Initial (Class II Myasthenia Gravis Foundation of America clinical classification)

	b. Second (Class III Myasthenia Gravis Foundation of America clinical classification)<br>c. Third (Class IV Myasthenia Gravis Foundation of America clinical classification)<br>d. Terminal (Class V Myasthenia Gravis Foundation of America clinical classification)<br>e. I do not know<br>f. I do not wish to answer
13. Which of the following is/are the most common symptom/s that you experience?	a. Muscle weakness<br>b. Blepharoptosis<br>c. Easy fatigue<br>d. Respiratory distress<br>e. Diplopia<br>f. Other
14. Do you have myasthenic crises?	a. Yes<br>b. No<br>c. I do not know/ I do not wish to answer
15. If you answered "a" in the previous question, how many episoded did you have in the last 3 months?	a. Number<br>b. I do not know/ I do not wish to answer
16. Do you take any medications for MG?	a. Yes<br>b. No<br>c. I do not wish to answer
17. If you answered "a" in the previous question, choose the appropriate answer	a. Only pyridostigmine<br>b. Pyridostigmine and cortisol<br>c. Pyridostigmine, cortisol and an immunosuppresant<br>d. Other<br>e. I do not know/ I do not wish to answer
18. Do you have any other chronic health conditions?	a. Yes<br>b. No<br>c. I do not know/ I do not wish to answer
19. Do you have a disability percentage greater than 50% as a result of MG?	a. Yes<br>b. No<br>c. I do not wish to answer
20. Gender	a. Male<br>b. Female<br>c. I do not wish to answer
21. Year of birth	Date
22. Marital status	a. Never married<br>b. Married<br>c. Divorced/separated<br>d. Widowed<br>e. I do not wish to answer
23. How many children do you have?	Open response
24. How many people do you currently live with?	Open response
25. Select your educational level	a. I have not finished primary school<br>b. Primary school<br>c. Secondary school<br>d. High school<br>e. College/Vocational training<br>f. University

	g. Postgraduate studies<br>h. I do not know/ I do not wish to answer
26. In which prefecture are you currently living in?	a. Open response<br>b. I do not wish to answer
27. What is your employment status?	a. Self-employed<br>b. Employee<br>c. Working without compensation in the family business<br>d. Retired<br>e. Unemplyed<br>f. Student/Housework<br>g. Other<br>h. I do not wish to answer
28. Which of the following better described your insurance coverage?	a. Public<br>b. Private<br>c. Private & public<br>d. No insurance<br>e. Coverage for indigent people<br>f. I do not wish to answer
29. Has your MG diagnosis affected your employment status?	a. No<br>b. Decreased hours<br>c. Changed job<br>d. I am not able to work due to MG<br>e. Retired due to disability
30. Which of the following better describes the economic status of your household?	a. Very severe financial challenges<br>b. Severe financial challenges<br>c. Some financial challenges<br>d. Good financial status<br>e. Very good financial status<br>f. Excellent financial status<br>g. I do not wish to answer
31. During the last 12 months, how greatly do you believe that the healthcare costs associated with MG affected your houshold economic status?	a. No effect<br>b. Minor effect<br>c. Some effect<br>d. Important effect<br>e. Very important effect<br>f. I do not know<br>g. I do not wish to answer
